# Fbxw7 regulates Notch to control specification of neural precursors for oligodendrocyte fate

**DOI:** 10.1186/1749-8104-7-15

**Published:** 2012-05-03

**Authors:** Julia L Snyder, Christina A Kearns, Bruce Appel

**Affiliations:** 1 Department of Biological Sciences, Vanderbilt University, Nashville, TN, USA; 2 Departments of Pediatrics and Cell and Developmental Biology, University of Colorado School of Medicine, Aurora, CO, USA; 3 University of Colorado School of Medicine, MS 8108, Aurora, CO, 80045, USA

**Keywords:** Notch, Glia, Oligodendrocyte, Myelin, Neural precursor, Zebrafish

## Abstract

**Background:**

In the developing vertebrate nervous system elevated levels of Notch signaling activity can block neurogenesis and promote formation of glial cells. The mechanisms that limit Notch activity to balance formation of neurons and glia from neural precursors are poorly understood.

**Results:**

By screening for mutations that disrupt oligodendrocyte development in zebrafish we found one allele, called *vu56*, that produced excess oligodendrocyte progenitor cells (OPCs). Positional cloning revealed that the *vu56* allele is a mutation of *fbxw7*, which encodes the substrate recognition component of a ubiquitin ligase that targets Notch and other proteins for degradation. To investigate the basis of the mutant phenotype we performed in vivo, time-lapse imaging, which revealed that the increase in OPC number resulted from production of extra OPCs by ventral spinal cord precursors and not from changes in OPC proliferation or death. Notch signaling activity was elevated in spinal cord precursors of *fbxw7* mutant zebrafish and inhibition of Notch signaling suppressed formation of excess OPCs.

**Conclusion:**

Notch signaling promotes glia cell formation from neural precursors in vertebrate embryos. Our data indicate that Fbxw7 helps attenuate Notch signaling during zebrafish neural development thereby limiting the number of OPCs.

## Background

Oligodendrocyte progenitor cells (OPCs), dividing migratory cells that differentiate as myelinating oligodendrocytes, arise from mitotically active neural precursors that occupy distinct regions of the central nervous system (CNS). In the spinal cord, most OPCs arise from the ventrally positioned pMN domain ( recursors of motor Neurons), which expresses the Olig1 and Olig2 transcription factors [1-3] and generates motor neurons prior to OPC formation [4-7]. Olig1 and Olig2 are necessary for development of both motor neurons and OPCs [4,5], therefore, regulatory mechanisms must exist to specify Olig1/2^+^ pMN precursors for either motor neuron or OPC fate. pMN precursors express Ngn1 and Ngn2 transcription factors during neurogenesis and downregulate them prior to gliogenesis [8], suggesting that Ngns inhibit OPC specification. On the other hand, spinal cord precursors initiate expression of transcription factors Sox9, NFIA and NFIB prior to gliogenesis and these factors promote timely formation of oligodendrocytes and astrocytes [9,10], raising the possibility that they contribute to a mechanism that causes neural precursors to switch from neuron to glial cell production.

Signaling mediated by Notch receptors also plays a major role in balancing production of neurons and glia. Generally, loss of Notch signaling in vertebrate embryos results in loss of neural precursors, formation of excess early-born neurons and a deficit of glial cells, including oligodendrocytes [11-17]. This occurs, in part, by elevated expression of the proneural genes *Ngn1* and *Ngn2*[18], which promote cell cycle exit and neurogenesis with the consequent loss of later-born glia. Conversely, Notch signaling activity can promote formation of glia in various contexts. In particular, transgene-driven expression of the constitutively active Notch intracellular domain (NICD) in zebrafish during neurogenesis blocked neuron formation and caused a nearly two-fold excess in OPCs, which appeared to arise only from pMN precursors and not from other neural precursors [15], indicating that the ability of Notch to promote OPC specification is limited to those precursors that express Olig1/2. Consistent with this, ectopic activation of Notch signaling in the chick spinal cord caused formation of ectopic OPCs only in combination with Olig2 [8]. How Notch activity is controlled to balance neuron and OPC specification remains poorly understood.

By screening for mutations that disrupt oligodendrocyte development we found one allele, *vu56*, that caused formation of excess OPCs in numbers similar to that produced by NICD expression. Positional cloning revealed that *vu56* disrupted *fbxw7*, which encodes the F-box substrate recognition subunit of an E3 ubiquitin ligase that targets specific proteins, including Notch, for degradation. Our in vivo, time-lapse imaging experiments show directly that the excess OPCs of *fbxw7*^*vu56*^ mutant zebrafish are produced by pMN precursors. Additionally, our gene expression and functional tests provide evidence that Notch signaling is the principal target of Fbxw7 in pMN precursors for OPC specification. Recent data obtained from mice with brain-specific deletions of *Fbxw7* indicate that Fbxw7-mediated regulation of Notch and c-Jun is required for neural precursor differentiation and neural cell survival [19,20] and that *Fbxw7* is required to promote neurogenesis and limit formation of astrocytes [21]. The work presented here now reveals that modulation of Notch signaling activity by Fbxw7 within neural precursors regulates specification of OPC fate.

## Results

### Mutation of *fbxw7* produces excess oligodendrocyte lineage cells

**Figure 1:**
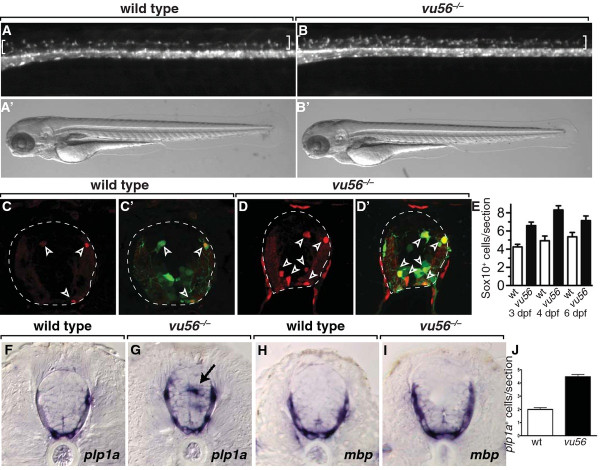
**The*vu56*mutation causes formation of excess OPCs.** (**A,B**) Lateral images of 3 dpf sibling and *vu56* mutants carrying the *Tg(olig2:EGFP)* reporter marking OPCs in dorsal spinal cord (brackets). Insets show bright field images of living larvae. (**C-D’**) Transverse sections of sibling and *vu56* mutant spinal cords processed for immunohistochemistry to detect Sox10 expression (red, arrowheads). Panels **C’** and **D’** show Sox10 labeling merged with *olig2:*EGFP (green). Dashed lines indicate outer edge of spinal cord. (**E**) Quantification of Sox10^+^ cells per spinal cord section in wild-type (wt) and *vu56* mutant larvae at 3, 4, and 6 dpf (n = 17 wild-type, 19 mutant at 3 dpf (*p*<0.0001), 7 wild-type and 10 mutant at 4 dpf (*p* = 0.0002), 5 wild-type and 5 mutant at 6 dpf (*p* = 0.0357)). (**F-I**) Transverse sections of 4 dpf wild-type sibling and *vu56* mutant larvae, at the level of trunk spinal cord, processed for in situ RNA hybridization to detect *plp1a* (**F,G**) and *mbp* (**H, I**) expression. Arrow indicates ectopic *plp1a* expression. (**J**) Quantification of *plp1a*^*+*^ oligodendrocytes per section in wild-type and *vu56* mutant larvae at 4 dpf (n = 4 larvae per genotype; *p*<0.0001). Error bars represent SEM.

By screening for changes in the number and distribution of oligodendrocyte lineage cells marked by *Tg(olig2:EGFP)* reporter gene expression, we identified a mutation designated *vu56*, which, when homozygous, resulted in excess EGFP^*+*^ dorsal spinal cord cells at 3 days post fertilization (dpf) (Figure 1A, B). Mutant larvae had no apparent morphological defects (Figure 1A’, B’) but did not inflate swim bladders, which are required for buoyancy and feeding, and died by 9 dpf. We verified that EGFP^+^ cells belonged to the oligodendrocyte lineage by performing immunohistochemistry to detect Sox10 protein, which marks both OPCs and myelinating oligodendrocytes in zebrafish [22]. All dorsal spinal cord EGFP^+^ cells in *vu56* mutant larvae were Sox10^+^, as were ventral EGFP^+^ cells that occupied positions characteristic of OPCs (Figure 1C-D’). Quantification revealed approximately 1.5 fold more Sox10^+^ cells per transverse section taken from *vu56* mutant larvae than wild-type larvae. This excess persisted through at least 6 dpf (Figure 1E). To learn if OPCs underwent differentiation we used in situ RNA hybridization to investigate expression of myelin genes. Wild-type larvae expressed *plp1a* and *mbp* near the pial surface of the spinal cord (Figure 1F, H). *vu56* mutant larvae also expressed *plp1a* and *mbp* near the pial surface and, occasionally, expressed *plp1a* at ectopic, medial spinal cord positions (Figure 1G, I). *plp1a*^+^ and *mbp*^+^ cells were also in excess number in *vu56* mutant larvae compared to wild type (Figure 1J and data not shown).

**Figure 2:**
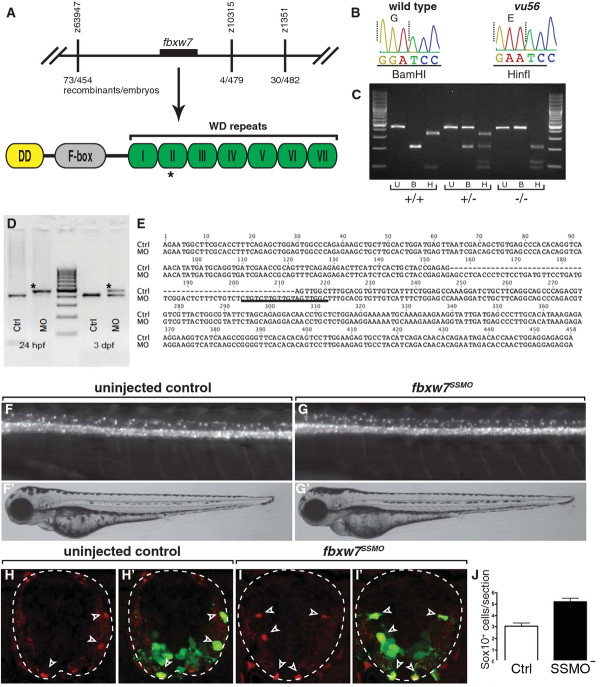
***fbxw7*is the gene mutated by the*vu56*allele.** (**A**) Schematic representation of genetic mapping and sequencing results. The *vu56* mutation mapped to a region of Chromosome 1 containing *fbxw7*. The *vu56* mutation created an amino acid substitution within the second WD repeat of the predicted Fbxw7 protein. (**B**) Sequence traces from wild-type and *vu56* mutant cDNAs. The *vu56* mutation changed a G to an A, converting a BamHI restriction site to a HinfI site. (**C**) RFLP genotyping of homozygous wild type (+/+), heterozygous (+/−) and homozygous mutant (−/−) larvae. U = uncut, B = BamHI digest, H = HinfI digest. (**D**) RT-PCR analysis of *fbxw7* mRNA splicing in control (Crtl) and *fbxw7*^*SSMO*^-injected (MO) 24 hpf embryos and 3 dpf larvae. Upper band in MO lanes (asterisks) indicates splice blocking products. (**E**) Sequence analysis of RT-PCR products from control embryos, which lack intron 4 sequence (dashed lines) and *fbxw7*^*SSMO*^-injected embryos, which retain intron 4 sequence. Underlined sequence is complementary to the splice-blocking morpholino. (**F,G**) Lateral images of 3 dpf uninjected control and *fbxw7*^*SSMO*^-injected larvae. Insets show corresponding bright field images. (**H-I’**) Transverse sections of 3 dpf uninjected control and *fbxw7*^*SSMO*^ morpholino injected larvae showing Sox10 expression (red, arrowheads). Panels **H’** and **I’** show Sox10 labeling merged with *olig2:*EGFP (green). (**J**) Quantification of Sox10^+^ cells per section in control and *fbxw7*^*SSMO*^-injected larvae (n = 8 larvae each genotype; *p* = 0.0001). Error bars represent SEM.

We used simple sequence length polymorphisms (SSLPs) to map the *vu56* mutation to a 0.6 cM region of chromosome 1, between the markers z63947 and z10315 (Figure 2A). This region included *fbxw7,* which we chose as a candidate because expression of human *FBXW7* was repressed in glioma [23,24]. Sequencing of PCR products amplified from genomic DNA of *vu56* mutant larvae revealed a missense mutation in exon 8 (Figure 2B) predicted to change a neutral glycine residue at amino acid position 261 to a negatively-charged glutamic acid residue near a critical substrate binding site within the second WD repeat (Figure 2A). Both SIFT [25] and PolyPhen [26] programs, which predict the functional effect of amino acid substitutions, identified this mutation as deleterious.

This missense mutation also created a Restriction Fragment Length Polymorphism (RFLP) by changing a BamHI site to a HinfI site. To test linkage between this mutation and the excess oligodendrocyte phenotype, we used the RFLP to genotype larvae produced by intercrosses of heterozygous adults. Whereas phenotypically wild-type larvae were either homozygous for the BamHI allele or heterozygous, all 70 larvae scored as mutant were homozygous for the HinfI allele (Figure 2C and data not shown).

To further validate our identification of *fbwx7* as the gene mutated by the *vu56* allele, we designed a morpholino oligonucleotide (*fbxw7*^*ssMO*^) to bind to the splice acceptor site of exon 4, which contains the F-box domain, with the expectation that it would alter RNA splicing thereby preventing translation of wild-type protein. RNA harvested from 24 hpf embryos and 3 dpf larvae injected with *fbxw7*^*ssMO*^ at one-cell stage produced RT-PCR products with larger molecular weights than control RNA (Figure 2D), suggesting that *fbxw7*^*ssMO*^ prevented excision of intron 3. We confirmed this by sequencing, which revealed introduction of a premature stop codon before the F-box domain (Figure 2E). We next injected different amounts of *fbxw7*^*ssMO*^ into newly fertilized *Tg(olig2:EGFP)* embryos. At a 2 ng dose, larvae appeared morphologically normal but had excess dorsal EGFP^+^ spinal cord cells compared to wild type (Figure 2F-G’). Quantification of Sox10^+^ cells revealed that the number of oligodendrocyte lineage cells in *fbxw7*^*ssMO*^ injected larvae was similar to that of *vu56* mutant larvae (Figure 2H-J). We conclude that loss of *fbxw7* function causes formation of excess oligodendrocytes in zebrafish and hereafter refer to the *vu56* allele as *fbxw7*^*vu56*^.

**Figure 3:**
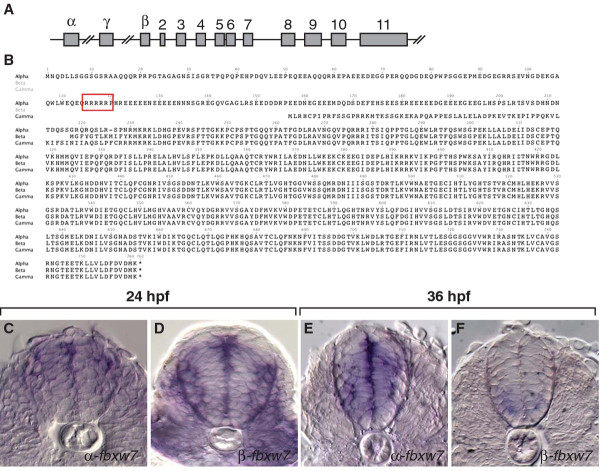
***fbxw7*gene structure and expression.** (**A**) Schematic representation of *fbxw7* gene structure. Three alternative first exons, α, γ, and β, are spliced to exons 2–11. (**B**) Predicted amino acid sequences of Fbxw7 isoforms. The putative nuclear localization sequence of the α isoform is boxed. (**C-F**) Transverse sections through the level of the trunk spinal cord showing α and β-specific RNA expression detected by in situ hybridization. At 36 hpf, when pMN precursors initiate OPC formation, medial spinal cord cells α-*fbxw7* at relatively high level whereas β-*fbxw7* transcripts are only evident at low level in ventral spinal cord.

Mammals express three Fbxw7 isoforms, which are produced by splicing of distinct first exons to a common sequence containing 10 exons (Figure 3A) [27]. Isoform-specific exons are transcribed by different promoters, resulting in distinct expression patterns. In adult mice, most tissues express α transcripts, brain expresses β transcripts at high level and γ transcripts are enriched in heart and skeletal muscle [28]. Encoded within isoform-specific exons are amino acid motifs that determine subcellular localization with α, β and γ isoforms localizing to nuclei, cytoplasm and nucleoli, respectively [29]. Using a combination of EST clones, PCR to amplify sequences predicted by Ensembl and 5′ RACE we identified α, β and γ transcripts in zebrafish embryos and used these to predict the encoded polypeptides (Figure 3B). The α-specific exon encodes a basic domain with similarity to Nuclear Localization Sequences whereas the β-specific exon, like that of the β exon of frogs [30], lacks sequence predicted to encode a hydrophobic transmembrane domain implicated in cytoplasmic localization of the mammalian β isoform [29].

We used in situ RNA hybridization to investigate isoform-specific expression in developing zebrafish embryos. Consistent with mammalian expression, somatic mesoderm, which gives rise to muscle, expressed high levels of γ transcripts whereas nervous system did not (data not shown). α transcripts were expressed throughout the brain and spinal cord and appeared to be maintained at particularly high levels in medial neural tube, consistent with expression by neural precursors (Figure 2C, E). Neural tissue also expressed β transcripts, but by 36 hpf expression appeared to be weak and limited to ventral spinal cord (Figure 3D, F).

### Loss of *fbxw7* function causes formation of excess OPCs from pMN precursors

**Figure 4:**
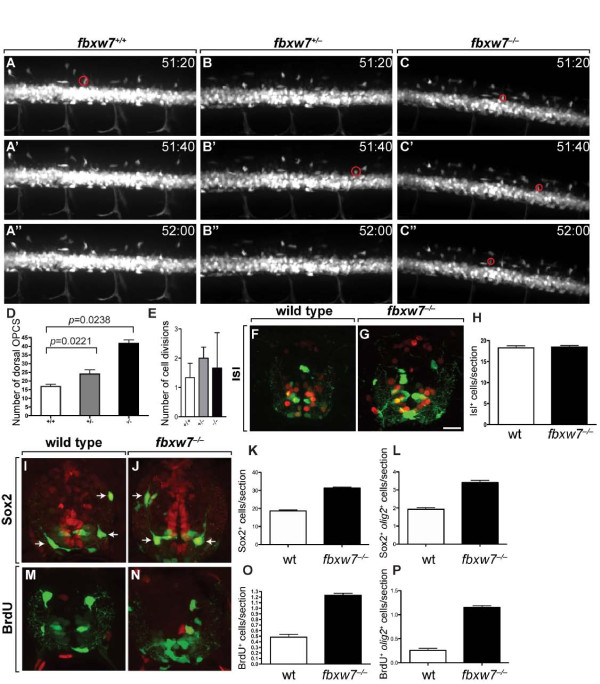
**Excess OPCs arise from ventral spinal cord precursors in*fbxw7***^***vu56***^**mutant larvae.** (**A-C”**) Images of spinal cords, from lateral orientation, captured from time-lapse movies of *fbxw7*^*+/+*^ (**A-A”**)*, fbxw7*^*+/−*^ (**B-B”**) and *fbxw7*^*−/−*^ (**C-C”**) larvae carrying the *Tg(olig2:EGFP*) transgene to mark migrating OPCs. Dorsal is up in all images. Numbers in each panel indicate time (hours, minutes) post fertilization. Red circles mark newly formed OPCs initiating migration from ventral spinal cord. (**D**) Quantification of OPCs that migrated dorsally in *fbxw7*^*+/+*^ (n = 6), *fbxw7*^*+/−*^ (n = 8) and *fbxw7*^*−/−*^ (n = 3) larvae from 48 to 63 hpf. (**E**) Average number of OPC divisions in each genotype. (**F,G**) Transverse sections of 3 dpf spinal cords labeled with anti-Isl antibody to mark motor neurons (red) in combination with *olig2:*EGFP expression (green). (**H**) Quantification of Isl^+^ cells per section in wild-type sibling and *fbxw7*^*−/−*^ larvae (n = 19 wild-type and 17 mutant larvae; *p* = 0.7658). (**I,J**) Transverse sections of 3 dpf spinal cords labeled with anti-Sox2 antibody to mark neural precursors (red) in combination with *olig2:*EGFP expression (green). Oligodendrocyte lineage cells also expressed Sox2 (arrows). Quantification of total spinal cord Sox2^+^ (**K**) and Sox2^+^*olig2:*EGFP^+^ (**L**) cells in wild-type and mutant larvae (n = 17 wild-type and 16 mutant larvae; *p*<0.0001). (**M,N**) Transverse sections of 3 dpf spinal cords labeled to detect BrdU incorporation (red) in combination with *olig2:*EGFP expression (green). Quantification of total spinal cord BrdU^+^ (**O**) and BrdU^+^*olig2:*EGFP^+^ (**P**) cells per section in wild-type sibling and *fbxw7*^*−/−*^ larvae (n = 18 wild-type and 15 mutant larvae; *p*<0.0001). Error bars represent SEM.

We identified the source of excess oligodendrocytes in mutant animals using time-lapse imaging. To minimize differences in OPC migration that might result from differences in developmental stage we mounted multiple embryos produced by matings of *fbxw7*^*vu56+/−*^*;Tg(olig2:EGFP)* adults in the same imaging chamber, sequentially collected time-lapse image sequences from individual embryos and genotyped them at the end of the imaging period. Between 48 and 63 hpf, the period of most ventral to dorsal spinal cord migration, an average of 17 OPCs occupied dorsal spinal cord between somites 5–9 in *fbxw7*^*+/+*^ embryos (Figure 4A-A”, D). During the same period, an average of 23 and 40 OPCs occupied dorsal spinal cord in *fbxw7*^*+/−*^ and *fbxw7*^*−/−*^ embryos, respectively (Figure 4B-C”, D). To learn if a change in OPC proliferation also contributed to the difference in OPC number, we counted divisions of dorsally migrating OPCs. Although we found a trend toward slightly more divisions in heterozygous and homozygous mutant larvae than in wild type, the differences did not reach statistical significance (Figure 4E). Our movies revealed no evidence of cells eliminated by apoptosis, which can be identified by cell fragmentation [31], indicating that differences in cell death do not account for differences in oligodendrocyte number. Therefore, we conclude that the excess oligodendrocytes of *fbxw7*^*vu56*^ mutant larvae result mostly from excess production of OPCs from ventral spinal cord precursors.

Ventral spinal cord precursors also produce motor neurons, consequently, one possible mechanism that could account for specification of excess OPCs is conversion of cells from motor neuron to OPC fate. To test this we counted motor neurons using immunohistochemistry. At 3 dpf, there was no apparent difference in the number of Isl^+^ motor neurons between *fbxw7*^*vu56*^ mutant larvae and their wild-type siblings, which would include *fbxw7*^*+/+*^ and *fbxw7*^*+/−*^ embryos (Figure 4F-H).

An alternative explanation for the formation of excess OPCs is that *fbxw7*^*vu56*^ mutant larvae have more spinal cord precursors at the time of gliogenesis. To test this we investigated expression of Sox2, a commonly used marker of neural precursors and neural stem cells [32]. By 3 dpf, Sox2 expression in wild-type larvae was mostly limited to cells that lined the central canal, located in ventral spinal cord, including *olig2*:EGFP^+^ pMN precursors (Figure 4I). By contrast, Sox2 was expressed at high level in cells along the entire medial septum and central canal of 3 dpf *fbxw7*^*vu56*^ mutant larvae (Figure 4J). Both the total number of Sox2^+^ cells and the number of Sox2^+^*olig2:*EGFP^+^ cells were increased about 1.7-fold in mutant larvae relative to wild-type larvae (Figure 4K,L). As an additional test of neural precursor number we labeled S-phase cells with a brief pulse of BrdU. 3 dpf *fbxw7*^*vu56*^ mutant larvae had more than twice the number of BrdU^+^ and BrdU^+^*olig2*:EGFP^+^ spinal cord cells than their wild-type siblings (Figure 4M-P). Therefore, *fbxw7*^*vu56*^ mutant larvae maintained excess number of dividing neural precursors, a subset of which produced excess OPCs.

### Fbxw7 limits OPC specification by limiting Notch signaling activity

**Figure 5:**
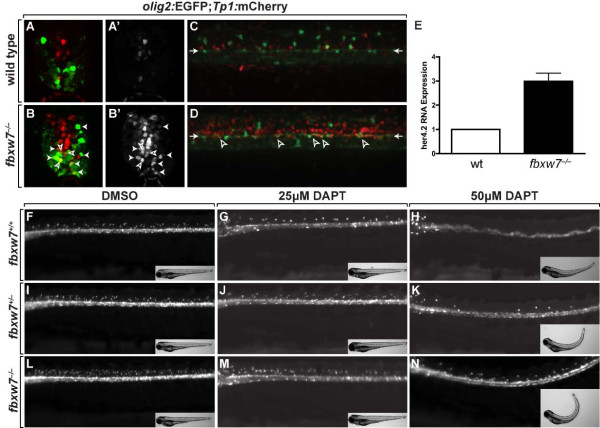
**Fbxw7 regulates oligodendrocyte number by regulating Notch signaling.** (**A,B**) Transverse sections of 3 dpf wild-type (**A**) and *fbxw7*^*vu56*^ mutant (**B**) spinal cords showing *Tp1*:mCherry and *olig2:*EGFP expression. Outlined arrowheads mark *Tp1*:mCherry^+^, *olig2:*EGFP^+^ precursors. Solid arrowheads indicate *Tp1*:mCherry^+^, *olig2:*EGFP^+^ oligodendrocyte lineage cells. (**A’,B’**) *Tp1*:mCherry images alone. (**C,D**) Confocal z stack projections, from lateral view, of *olig2:*EGFP and *Tp1:*mCherry in 6 dpf wild-type sibling and *fbxw7*^*vu56*^ mutant larvae. Arrowheads point to row of *olig2:*EGFP^+^ precursors lining the central canal. Outlined arrowheads mark *Tp1*:mCherry^+^, *olig2:*EGFP^+^ precursors. (**E**) Relative levels of *her4.2* mRNA in 4 dpf wild-type and *fbxw7*^*vu56*^ mutant larvae determined by quantitative PCR. Wild-type level was assigned an arbitrary level of 1.0 (*p* = 0.0272). (**F-N**) Lateral images of living 3 dpf larvae treated with DMSO or DAPT plus DMSO from 36–48 hpf. All larvae carried the *Tg*(*olig2:EGFP*) reporter to mark OPCs and oligodendrocytes in dorsal spinal cord. DAPT reduced the number of oligodendrocyte lineage cells in larvae of each genotype. Insets show bright field images of larvae. Each larva was genotyped following the experiment.

Expression of constitutively active Notch1a prior to OPC specification produced an increase in the number of oligodendrocytes nearly identical to that of the *fbxw7*^*vu56*^ mutation [15]. The signaling active intracellular domain of Notch1 is targeted for degradation by Fbxw7-mediated ubiquitination [33-35]. Therefore, the excess OPCs in *fbxw7*^*vu56*^ mutant larvae could result from elevated Notch signaling. As a test of this possibility, we investigated expression of the Notch reporter transgene *Tg(Tp1bglob:hmgb1-mCherry)*, which drives mCherry expression (hereafter *Tp1*:mCherry) using a regulatory element containing RBP-Jκ binding sites [36]. At 3 dpf, *Tp1*:mCherry expression was evident in a few cells near the central canal and medial septum of wild-type larvae and only apparent at low level in *olig2*:EGFP^+^ cells located away from the spinal cord midline (Figure 5A, A’). By contrast, numerous cells lining the central canal and medial septum of *fbxw7*^*vu56*^ mutant larvae expressed *Tp1*:mCherry at high level (Figure 5B, B’). Additionally, weaker *Tp1*:mCherry signal was evident in numerous cells away from the midline, including *olig2*:EGFP^+^ oligodendrocyte lineage cells. At 6 dpf, confocal microscope images of wild-type larvae viewed from lateral orientation revealed mCherry expression in scattered cells of the spinal cord (Figure 5C). *olig2:*EGFP^+^ radial glial precursors, which persist into adulthood [37], were evident as a row of elongated cells at the level of the spinal cord central canal. Few of these cells expressed mCherry, indicating that they had little Notch signaling activity. In *fbxw7*^*−/−*^ larvae, numerous spinal cord cells expressed mCherry, including *olig2:*EGFP^+^ radial glial precursors (Figure 5D). We also investigated expression of the Notch target gene *her4.2* using quantitative RT-PCR and found that RNA levels were approximately 3-fold higher in *fbxw7*^*−/−*^ larvae than wild-type larvae at 4 dpf (Figure 5E). Therefore, Notch signaling activity is elevated in the absence of *fbxw7* function, consistent with the possibility that Fbxw7 targets Notch for degradation in the zebrafish nervous system.

If elevation of Notch signaling accounts for the excess oligodendrocytes of *fbxw7*^*vu56*^ mutant larvae, then inhibition of Notch signaling should suppress the mutant phenotype. To test this we used the γ-secretase inhibitor *N*-*N*-(3,5-difluoro- phenacetyl-l-alanyl)]-*S*-phenylglycine *t*-butyl ester (DAPT), which prevents release of the signaling-active Notch intracellular domain and produces phenotypes in zebrafish embryos that are indistinguishable from Notch pathway mutations [38]. We applied DAPT to embryos produced by intercrosses of *fbxw7*^*+/−*^ adults from 36–48 hpf, the time at which OPCs are specified from neural precursors, assessed the number of dorsally migrated OPCs revealed by *Tg(olig2:EGFP)* reporter expression at 72 hpf and genotyped each animal. When exposed to 25 μM DAPT, larvae of all three possible genotypes (*fbxw7*^*+/+*^*, fbxw7*^*+/−*^*, fbxw7*^*−/−*^) had normal morphologies and slightly fewer dorsal OPCs than the corresponding genotypes treated only with DMSO (Figure 5G, J, M). 50 μM DAPT frequently produced pronounced morphological defects, including upwardly curled tails, and blocked formation of most OPCs in *fbxw7*^*+/+*^ and *fbxw7*^*+/−*^ larvae (Figure 5H, K), consistent with our previous demonstration that Notch signaling is necessary for OPC specification [15]. We found two classes of phenotype among 50 μM DAPT-treated *fbxw7*^*−/−*^ larvae. 25% of the homozygous mutant larvae (n = 12) had morphological defects and fewer OPCs than control treated *fbxw7*^*+/+*^ larvae (Figure 5N). The remaining homozygous mutant larvae had apparently normal morphologies and approximately the same number OPCs as control treated *fbxw7*^*+/+*^ larvae (data not shown). Therefore, inhibition of Notch signaling suppresses the excess OPC phenotype of *fbxw7*^*vu56*^ mutant larvae, supporting our hypothesis that Fbxw7 limits the number of OPCs specified from neural precursors by regulating the amount of actively signaling Notch proteins.

## Discussion

During development, dividing neural precursors of the ventral spinal cord give rise first to motor neurons and then oligodendrocytes. The orderly formation of motor neurons and oligodendrocytes requires mechanisms that both maintain dividing neural precursors through neurogenesis and switch them from producing neurons to oligodendrocytes. Notch signaling is essential for balancing neural precursor maintenance with motor neuron and oligodendrocyte specification because mutations that disrupt Notch signaling cause loss of neural precursors, formation of excess early born neurons and a deficit of oligodendrocytes [15]. How Notch signaling is controlled to maintain a precise balance of precursors, neurons and oligodendrocytes is not known. Our work now indicates that negative regulation of Notch activity by Fbxw7 ubiquitin ligase limits the number of neural precursors specified for oligodendrocyte fate.

The founding member of metazoan *Fbxw7* genes is nematode *sel-10*, which was identified as a negative regulator of the *Notch* homolog *lin-12*[39]. Work since then has shown that sel-10/Fbxw7 is the substrate recognition subunit of a SCF ubiquitin ligase that targets Notch proteins for degradation [33-35], as well as several other proteins that are important for cell cycle control [40]. Accordingly, mutation of human *FBXW7* has been implicated in numerous cancers [40,41]. Substrate recognition by Fbxw7 is dependent upon phosphorylation of specific amino acid sequences called phosphodegrons [40]. The Notch phosphodegron lies within the NICD [42], which is responsible for Notch signal transduction, thereby providing a mechanism for attenuation of Notch signaling activity.

In a previous study we showed that forced expression of NICD in transgenic zebrafish blocked formation of most neurons and produced an excess of OPCs [15] nearly identical to that of *fbxw7* mutants. These data implied that Notch signaling must be restricted to properly balance neuron and OPC formation but the mechanisms that attenuate Notch signaling in the nervous system were not clear. The work reported here showed that Notch activity was elevated in neural precursors of *fbxw7* mutants and that inhibition of Notch signaling suppressed formation of excess OPCs, indicating that Fbxw7 limits Notch signaling within spinal cord precursors to limit OPC production. These data therefore contribute to growing evidence that Notch activity can be regulated at numerous levels, including at the level of signal attenuation via ubiquitin ligase-mediated degradation [43-46].

Our in vivo time-lapse imaging showed directly that pMN precursors produced excess OPCs in *fbxw7* mutant zebrafish embryos. Our data therefore strongly indicate that Fbxw7 controls OPC number by regulating Notch signaling activity in neural precursors. We have previously shown that individual pMN precursors can produce both motor neurons and OPCs in zebrafish [7] and that a deficit of neurons accompanied the formation of excess OPCs resulting from transgene-driven expression of NICD [15]. These data were consistent with a model in which Notch functions as a binary switch, with high signaling levels blocking neurogenesis and promoting oligodendrogenesis. However, we found no decrease in the number of motor neurons in *fbxw7* mutant embryos, despite elevated levels of Notch signaling. This raises the possibility that subtle differences in Notch activity can have distinct effects on the fates of neural precursors, consistent with observations that both fewer and excess copies of Notch pathway genes produce mutant phenotypes in flies and vertebrates [47]. Our in situ RNA hybridization experiments revealed no evidence of temporally and spatially regulated *fbxw7* expression that could account for a fine-scale modulation of Notch activity in neural precursors. Therefore, the phosphorylation status of Notch phosphodegrons might be a key feature in regulation of pMN precursors for oligodendrocyte fate.

Two recent investigations of mice in which transgenic expression of Nestin-Cre was used to eliminate Fbxw7 from neural precursors also produced evidence that Notch is an important target of Fbxw7, but additionally arrived at some conclusions that were different from each other and from our own. In one report, Hoeck et al. described mutant mice that that had reduced cellularity throughout the brain, which they attributed to a failure of neural stem cell differentiation and neurogenesis due to elevated Notch signaling, and to elevated neuronal progenitor death, resulting from elevated levels of c-Jun, a known Fbxw7 target [20]. This group reported no difference in the number of cells that expressed markers of astrocytes and oligodendrocytes in E18.5 mutant brains or in neurospheres produced from mutant animals. A second report also described evidence of elevated Notch signaling and reduced neurogenesis, but in contrast did not find an increase in cell death or c-Jun protein levels [21]. Furthermore, Matsumoto et al. documented excess numbers of cells that expressed the astrocyte marker GFAP, which was suppressed in culture by inhibition of Notch signaling but, in contrast to our own work, found no change in the numbers of oligodendrocyte lineage cells in mutant P0.5 mutant brains or in cell culture.

One possible explanation for the apparently different effects of *Fbxw7* mutation in mice and zebrafish is that zebrafish might have a second *fbxw7*-like gene, which could partially compensate for the loss of function caused by the *vu56* mutant allele. However, our queries of the zebrafish genome did not reveal evidence of a duplicated gene. A second possibility is that the relatively small number of OPCs and the ability to observe them directly in zebrafish revealed changes in cell number that were obscured in the mouse studies. OPC number is regulated by cell density in vitro [48] and by limiting amounts of growth factor [49,50] and cell-cell contact in vivo [51]. Consequently, regulation of OPC proliferation following their formation in *Fbxw7* mutant mice or cell culture might have resulted in normal numbers at the time points chosen for analysis. A third possibility is that different types of *Fbxw7* mutant alleles might produce different phenotypes. Whereas the mouse alleles were designed to eliminate the F-box domain, the zebrafish *vu56* allele is a missense mutation within the WD substrate recognition domain. The majority of human *FBXW7* alleles implicated in cancer are missense mutations [41] and a missense mutation created in mouse *Fbxw7* was functionally distinct from a null allele [52]. However, a splice-blocking antisense morpholino designed to cause truncation of zebrafish Fbxw7 before the F-box produced a phenotype similar to the *vu56* missense allele, although this did not produce a null effect because splicing was not completely blocked.

Regulation of OPC and astrocyte number via Fbxw7-mediated inhibition of Notch activity is consistent with other studies that implicated a broad role for Notch signaling in gliogenesis. For example, addition of soluble Notch ligand to neural cell cultures enhanced production of astrocytes [53,54] and Notch signaling directly promoted transcription of the radial glia and astrocyte-associated genes *BLBP* and *GFAP*[53,55]. In vivo, transient expression of Notch ligands by newly specified neurons appeared to activate Notch signaling in neighboring precursors, driving them toward astrocyte fate via activation of nuclear factor I (NFI) expression and demethylation of astrocyte specific genes [56]. Expression of NICD or the Notch effector Hey2 (also known as Hesr2) drove formation of Müller glia in zebrafish and mouse retinas [57,58] and NICD produced excess radial glia in mouse forebrain [59]. Although the mechanistic details of oligodendrocyte specification are still poorly understood, Notch signaling similarly promoted formation of oligodendrocytes in zebrafish [15], at least in part by regulating *cyclin-dependent kinase inhibitor 1c* activity [60]. Our work presented here now provides evidence that Fbxw7 limits the number of OPCs formed by neural precursors by attenuating Notch signaling, thereby opening a new avenue to understanding the mechanisms that specify oligodendrocyte development.

## Conclusions

In this paper we show that mutation of *fbxw7*, which encodes a subunit of a ubiquitin ligase, resulted in production of excess oligodendrocyte progenitor cells from neural precursors in zebrafish embryos. Notch signaling was elevated in *fbxw7* mutant embryos and pharmacological inhibition of Notch signaling suppressed formation of excess oligodendrocyte progenitors indicating that Notch proteins are functionally relevant targets of Fbxw7-mediated ubiquitination during oligodendrocyte specification. Our data provide evidence that negative regulation of Notch activity by protein degradation controls production of appropriate numbers of myelinating glial cells from dividing neural precursors in vertebrate embryos.

## Methods

### Zebrafish husbandry

Embryos were produced by pair wise matings, raised at 28.5°C in egg water or embryo medium (EM), and staged to hours post-fertilization (hpf) or days post-fertilization (dpf) as previously described [61]. Zebrafish strains used include: AB, *Tg*(*olig2:EGFP*) [62], *Tg*(*Tp1bglob:hmgb1-mCherry*)^*jh11*^[36] and *fbxw7*^*vu56*^. All experiments were approved and conducted in accordance with the guidelines set forth by the Institutional Animal Care and Use Committee of the University of Colorado Denver, Anschutz Medical Campus.

### Mutant screen

The *vu56* allele was identified in a screen for mutations that altered the number and distribution of OPCs, revealed by *Tg(olig2:EGFP)* reporter expression. AB males were mutagenized with *N-*ethyl *N-*nitrosourea (ENU) as described previously [63]. Mutagenized males were crossed to *Tg(olig2:EGFP)* females to create an F1 generation. F1 fish were raised to adulthood and crossed to wild-type *Tg(olig2:egfp)* fish to create F2 families. F2 siblings were randomly intercrossed and their progeny screened using fluorescent stereomicroscopes. Identified F2 *vu56* heterozygotes were outcrossed to AB fish to propagate the line and to the WIK laboratory strain to create families for genetic mapping. The *vu56* allele has been maintained by repeated outcrossing to AB fish.

### Mapping and PCR genotyping

*fbxw7*^*vu56*^ mutants were identified at 3 dpf and collected with wild-type siblings for isolation of DNA in lysis buffer (10 mM Tris pH 8.0, 50 mM KCl, 0.3% Tween-20, 0.3% NP-40) with 1 μg/μL Proteinase K at 55°C overnight. Pooled DNA was used for bulked-segregant analysis [64] with published simple sequence length polymorphisms (SSLPs) (http://www.zfin.org). Individual embryos were used to determine recombination frequencies for finer mapping of *fbxw7*^*vu56*^. The following primers were designed to amplify sequences flanking the mutation for restriction fragment length polymorphism genotyping: *fbxw7* forward primer: 5′-CAG TTG ATT TAC CTT TGC GT-3′; reverse primer: 5′-TGT GTC AAT GTG TTT CGG TT-3′. Products were digested with BamHI and HinfI and analyzed using agarose gel electrophoresis.

### Isoform cloning and RT-PCR analysis of expression

We obtained clones corresponding to *fbxw7* α, β and γ transcripts using PCR to amplify genomic sequences, which were cloned into pCR2.1-TOPO vectors. The following primer pairs were used for amplification: α, 759 bp product, 5′-CAGAATGCCAAGTCCTTGTC-3′/5′-CCTATTCGGTGAGCGAAGG-3′; β, 254 bp product, 5′-GGCTCAGTCAGTCCGCTCAG-3′/5′-TTTATAGAAGATCATCTTTAAAGTG-3′; γ, 520 bp product, 5′-GCTTGGTGTGAACACTTAAAAC-3′/5′-CATAATTGCATCATTTCCACATT-3′. To investigate expression at different developmental stages we used the isoform specific forward primers with the reverse primer 5′-CGT CGT CTC TGT GGA ACC-3′ from the common region to amplify cDNA from single-cell, 24 hpf, and 3 dpf embryos.

### Immunohistochemistry

Embryos were fixed using 4% paraformaldehyde, embedded, frozen and sectioned using a cryostat microtome as previously described [15]. We used the following primary antibodies: rabbit anti-Sox10 (1:500) [60], mouse anti-Isl (39.4D5, Developmental Studies Hybridoma Bank (DSHB) Iowa City, Iowa USA, 1:100), mouse anti-BrdU (G3G40, DSHB, 1:10), and anti-Sox2 (ab97959, Abcam, 1:1000). For fluorescent detection of antibody labeling, we used Alexa Fluor 568 goat anti-mouse or goat anti-rabbit conjugates (Invitrogen, 1:200). Images were captured using either a Zeiss Axiovert 200 inverted microscope equipped with a PerkinElmer Ultraview ERS Live Cell Imager spinning disc confocal system or a Zeiss AxioObserver inverted microscope equipped with a PerkinElmer Ultra*VIEW* VoX confocal system and analyzed with Volocity software (PerkinElmer) and Adobe Photoshop. Image adjustments were limited to contrast enhancement, level settings, auto tone and cropping.

### In situ RNA hybridization

The following RNA probes were generated for this manuscript: *fbxw7*, which recognizes all isoforms, from an EST (EB835996), *α-fbxw7β-fbxw7*, and *γ-fbxw7* isoform specific probes from 24 hpf cDNA, and *plp1a* and *mbp*[65]. *in situ* RNA hybridization was performed according to published methods [66]. Embryos were either mounted for whole-mount imaging or embedded and sectioned as above. Images were captured using either an Olympus AX70 microscope equipped with DIC optics, a Retiga Exi-cooled CCD camera (QImaging) and Openlab software (Improvision) or a similarly equipped Zeiss AxioObserver inverted microscope and Volocity software (Improvision). Image data were exported to Adobe Photoshop and adjustments were limited to level settings, color balance and cropping.

### Quantitative PCR

RNA was isolated from 3 sets of 20 pooled wild-type larvae and 3 sets of 20 pooled *fbxw7*^*−/−*^ larvae at 4 dpf. Reverse Transcriptase was performed using Superscript III First Strand Synthesis for qPCR (Invitrogen). Real-time qPCR was performed on each sample in triplicate using an Applied Biosystems StepOne Plus machine and software version 2.1. Taqman Gene Expression Assays (Applied Biosystems) were used to detect *her4.2* (Dr03160688_g1) and *bactin1* (Dr03432610_m1).

### In vivo time-lapse imaging

Embryos were lightly anesthetized using Tricaine, mounted on their sides in 0.7% low-melting temperature agarose in 35 mm glass bottom dishes and covered with EM containing Tricaine. Z-stack images were captured every 15 minutes for 24 hours using an inverted Zeiss AxioObserver equipped with motorized and heated stage and a PerkinElmer Ultra*VIEW* VoX confocal system. The imaging chamber was maintained at 28.5°C. 4D data sets were analyzed using Volocity software (PerkinElmer) and movies were exported to QuickTime. Image adjustments were limited to contrast enhancements and cropping frame size.

### Morpholino injections

*fbxw7*^*SSMO*^, consisting of the sequence 5′-GCCAACTACAACAAGACAGAGACAG-3′ (Gene Tools, LLC) was designed to have sequence complementary to the boundary of intron 4 and exon 5 of *fbxw7*. The MO was resuspended in sterile water to a stock concentration of 1 mM and stored at room temperature. The stock was diluted in 2X injection buffer (240 mM KCl, 40 mM HEPES, and 0.5% Phenol red) to a concentration of 0.125 mM and 2 nL was injected into the yolk of one- to two-cell stage embryos.

### DAPT treatments

The γ-secretase inhibitor *N*-[*N*-(3,5-difluoro- phenacetyl-l-alanyl)]-*S*-phenylglycine *t*-butyl ester (DAPT) (Calbiochem) was resuspended in dimethyl sulfoxide (DMSO) to a stock concentration of 20 mM and stored in aliquots at −20°C. Embryos were manually dechorionated at 36 hpf and placed in 25 μM or 50 μM in EM with 1% DMSO for 12 hours at 28.5°C. DAPT solution was replaced with EM and embryos were allowed to develop to 3 dpf at 28.5°C. At 3 dpf embryos were imaged individually using a stereomicroscope equipped with bright field and epifluorescence optics followed by DNA isolation and *fbxw7*^*vu56*^ genotyping as described above.

### BrdU labeling

Dechorionated embryos were labeled with 5-bromo-2′-deoxyuadine (BrdU) (Roche) by incubating them in 20 mM BrdU in EM with 10% DMSO at room temperature for 30 min. The embryos were then rinsed and maintain at room temperature for 30 min and then fixed using 4% paraformaldehyde. After embedding and sectioning as described above, the tissue sections were treated with 2 N HCl for 30 min before processing for anti-BrdU immunohistochemistry.

### Data quantification and statistical analysis

Cell counts were obtained by direct observation of sections using the microscopes described above. For Sox10, *mbp*, and *plp1a* quantification, 10 sections per embryo were counted to produce the average number per section. For Isl quantification, 9 sections per embryo were counted to produce the average number per section. GraphPad Prism software was used for statistical analysis.

## Abbreviations

OPCs = Oligodendrocyte progenitor cells; CNS = Central nervous system; NICD = Notch intracellular domain; dpf = Days post fertilization; hpf = Hours post fertilization; SSLPs = Simple sequence length polymorphisms; PCR = Polymerase chain reaction; RFLP = Restriction fragment length polymorphism; MO = Morpholino oligonucleotide; ng = Nanogram; EST = Expressed sequence tag; PCR = Polymerase chain reaction; RACE = Rapid amplification of cDNA ends: DAPT, N-[N-(3,5-difluoro- phenacetyl-l-alanyl)]-S-phenylglycine t-butyl ester; DMSO = dimethyl sulfoxide; bp = Base pairs.

## Competing interests

The authors declare that they have no competing interests.

## Authors’ contributions

JLS performed all the experiments described in this manuscript except for quantitative PCR and Sox2 and BrdU immunohistochemistry, which was performed by CAK. BA conceived of the study and helped design and interpret experiments. JLS and BA wrote the manuscript. All authors read and approved the manuscript.
